# The world of contraception: A global mapping project across FIGO member societies

**DOI:** 10.1002/ijgo.70254

**Published:** 2025-06-02

**Authors:** Mikaela R. Koch, Pragya Rimal, Gesenia Echeverria, Meredith Robrahn, Aparna Sridhar

**Affiliations:** ^1^ David Geffen School of Medicine University of California Los Angeles California Los Angeles USA; ^2^ University of California Los Angeles Fielding School of Public Health California Los Angeles USA; ^3^ University of California Los Angeles California Los Angeles USA; ^4^ Department of Obstetrics and Gynecology University of California Los Angeles California Los Angeles USA

**Keywords:** collaboration, contraception, family planning, FIGO, international, member societies

## Abstract

**Objective:**

The International Federation of Obstetrics and Gynecology (FIGO) is positioned to work globally to improve contraceptive access and coverage; however, a lack of awareness regarding the contraceptive work conducted across its 139 professional member societies remains a concern. This study aimed to map the contraceptive landscape and programming across obstetric and gynecologic (OBGYN) member societies providing insights into the contraceptive work, challenges, and needs globally and identifying avenues for international collaboration.

**Methods:**

This was a qualitative study utilizing a 17‐item semi‐structured interview guide among FIGO member societies. Purposive sampling and reputational case selection were used to identify interviewees. Interviews were conducted in English, Spanish and French, translated and transcribed by external consultants, and thematically analyzed using Dedoose software.

**Results:**

Seventy member societies were included in the study and participation was well‐distributed across all five FIGO regions. Among respondents, 19% reported their society had a specific and dedicated working group focused on contraception while 84% had national contraceptive guidelines. The majority indicated their priorities lay in training physicians in contraceptive skills and counseling, adolescent contraception, and addressing misinformation and cultural barriers. Moreover, respondents also stated they would be most interested in collaborative efforts focused on provider training, regional collaboration, community awareness, and guideline development.

**Conclusion:**

Despite utilizing stakeholders who may not represent the full extent of society contraceptive programming, this study's strength lies in its large regional representation and sample size. Future efforts include strengthening regional collaboration, developing a global family planning network under FIGO and enhancing research and capacity building efforts.

## 1 INTRODUCTION

Maternal mortality remains one of the most devastating and preventable causes of global death with 99% occurring in developing countries.^1^ Contributing to this mortality are high risk pregnancies, abortion‐related deaths and short inter‐pregnancy intervals which remain a significant risk factor for both maternal and neonatal morbidity—exacerbating conditions such as maternal anemia and preterm birth.^2^, ^3^, ^4^ Compounding this, there remains a large unmet need for contraception which if addressed, studies show, could reduce maternal mortality by as much as 30%.^5^, ^6^ Contraception is thus a key healthcare intervention of our era, offering opportunities to enhance obstetric, maternal, reproductive, and general health efforts globally.

The contraceptive workforce, including obstetrician and gynecologists (OBGYN), primary care providers, nurses, midwives, and community healthcare workers, play a crucial role in delivering contraceptive services and ensuring accessible and comprehensive reproductive healthcare.^7^ The International Federation of Obstetrics and Gynecology (FIGO) is the world's largest alliance of national OBGYN societies with a mission to improve reproductive health.^8^ And yet, there remain challenges to global contraceptive efforts including political and cultural heterogeneity and language and religious barriers.^9^, ^10^, ^11^, ^12^, ^13^ These obstacles may complicate efforts by major international institutions, such as FIGO, to integrate family planning into national infrastructure and foster collaborative international efforts.

Given FIGO's aim of leveraging their global network to facilitate knowledge sharing and support initiatives to improve women and girls' health, there remains an opportunity for leadership in fostering international collaboration within family planning.^8^ OBGYN's across country‐led member societies engage in a variety of contraceptive initiatives; however, to date, no study has been done to understand this contraceptive landscape.

As the landscape of reproductive health evolves globally with changing laws, new humanitarian crisis, and novel contraceptive methods emerging on the market, sharing experiences and strategies is crucial—allowing countries to learn from one another's accomplishments and setbacks, avoid duplicating efforts, and adapt interventions to cultural and medical contexts. In a world that constantly pulls at our attention with competing resources, establishing a community of best practices through international cooperation is essential for making a global impact in reproductive health.

It is in this context that this study aimed to: (1) Provide insights into the contraceptive efforts done within OBGYN member societies (e.g postpartum IUD or safe abortion initiatives, sexual education campaigns in high schools, or research on the Nexplanon implant). (2) Identify challenges faced by member societies in conducting such contraception work. (3) Assess needs globally and identify avenues for international collaboration in this space.

## 2 MATERIALS AND METHODS

This was a qualitative study using semi‐structured interviews among individual members of FIGO ‐associated OBGYN societies. The study received IRB exemption status from the University of California Los Angeles (#23‐000966).

### 2.1 Research team

The principal investigator was AS, obstetrician and former chair of FIGO's contraception committee. Interviewers for this study included MK, GE, and MR. Lead interviewer (MK) ‐ who conducted all English interviews ‐ was a medical student at the time of the study with experience and past research focused on global women's health and family planning and entering the field of OBGYN. Other interviewers (GE and MR) were local undergraduate students selected for their passion for reproductive health and fluency in their respective languages (Spanish and French). PR was selected as the team's qualitative expert undergoing a PhD in Public Health at the time of the study with extensive experience with qualitative interviews. The entire research team was female.

### 2.2 Participant recruitment

Purposive sampling and reputational case selection were used, with recruitment based on both involvement within the member society and demonstrated commitment or expertise regarding contraception and sexual and reproductive health work. Standardized emails (Supporting Information S1) were sent (AS, MK) to all contacts listed on the FIGO website and society specific websites with follow‐ups sent roughly one week apart. Following the initial round of emails, key stakeholders and personal contacts were asked to provide additional contacts. Moreover, after each interview, participants were asked if they had contacts in neighboring countries. This was completed until 50% of member societies had been interviewed with distribution across all FIGO regions at which point further recruitment emails were paused.

Prior to the interview itself it was made clear to study participants that the research was supported by FIGO's contraception committee, and that AS was the former chair of the committee. Otherwise, interviewers themselves were identified only as research assistants and no further details about them were known. There was no former relationship established between interviewers and interviewees prior to study commencement.

### 2.3 Inclusion criteria

All 139 member societies listed on FIGO's website (https://www.figo.org/figos‐member‐societies) as of September 2023 were eligible to participate. Regional distribution was determined by reported membership with FIGO Allied Regional Federations and geographic distribution (https://www.figo.org/figo_council).

### 2.4 Exclusion criteria

There were no exclusion criteria. However, societies were no longer contacted if after three email requests there was no response, if we were unable to identify a viable email for the society, or if after multiple follow‐ups the individual did not respond for interview scheduling. There were no individuals that refused to participate.

### 2.5 Sample size

The initial goal of the study was to reach 50% of all FIGO member societies with proportionate distribution across the five FIGO regions.

### 2.6 Interview guide

A 17‐question interview guide was developed by PR, AS and MK with support from internal qualitative experts and stakeholders within global health including a final review by the FIGO Contraception Committee (Supporting Information S2). The objective of the interview guide was to obtain a sense of the local contraceptive landscape, society infrastructure, contraceptive programming, and future directions and areas for collaboration. Interview guides were revised (PR, MK) after the first two interviews based on reflections from the research team. Revisions included wording adjustments to make questions more open ended and question order for improved interview flow. The final interview guide was then translated into French and Spanish by consultants (Supporting Information S3 and S4). Interviewers were trained on the interview guide, having multiple weeks to review the script and undergoing practice interviews with team members.

### 2.7 Data collection

Interviews were conducted via Zoom and audio recorded after obtaining verbal consent from participants. There were no repeat interviews. Interviews were conducted in English, French and Spanish (MK, GE, MR). Interviews lasted up to 60 min. The majority of interviews were conducted with one individual; however, society requests to include multiple people on the call, in an attempt to provide a more comprehensive picture of society activity, were accommodated up to a total of four interviewees. When available, AS would join the Zoom to introduce the project and thank the interviewee for their participation, but was otherwise absent from the interview itself. There were no field notes taken during the interviews, however all interviews were transcribed verbatim as indicated below.

Data collection was halted once 50% of FIGO member societies had been contacted . The research team felt comfortable doing so having met initial study goals, and that, per discussions with interviewers, thematic saturation had been achieved.

### 2.8 Translation and transcription

English interviews were transcribed by the research team (MK). French and Spanish interviews were translated and transcribed by external consultants. All interviews were transcribed verbatim. Interviewees were welcome to review and correct the transcripts, although none requested to do so.

### 2.9 Data storage

Deidentified transcripts were stored in secure, password protected, HIPPA compliant, online platforms (Box, Dedoose) and accessible only to IRB approved research members.

### 2.10 Statistical analysis

Quantitative data was assessed using descriptive analysis for reporting frequencies. Qualitative data was coded using Dedoose software. A code book was created and agreed upon by the research team after three iterations of the codebook (PR, MK, AS) (Supporting Information S5). Utilizing a framework analysis, codes were categorized by research question, and subsequent codes were added to reflect themes throughout the transcripts. Two coders (MK and PR) coded the transcripts. Coders double coded 15% of one another's assigned transcripts until consensus was achieved in order to establish inter‐rater reliability. After each transcript, the coder generated a memo summarizing key themes from the interview. Coders met weekly to discuss and address discrepancies. Participants did not review results or provide feedback on findings during the course of the study.

## 3 RESULTS

**FIGURE 1:**
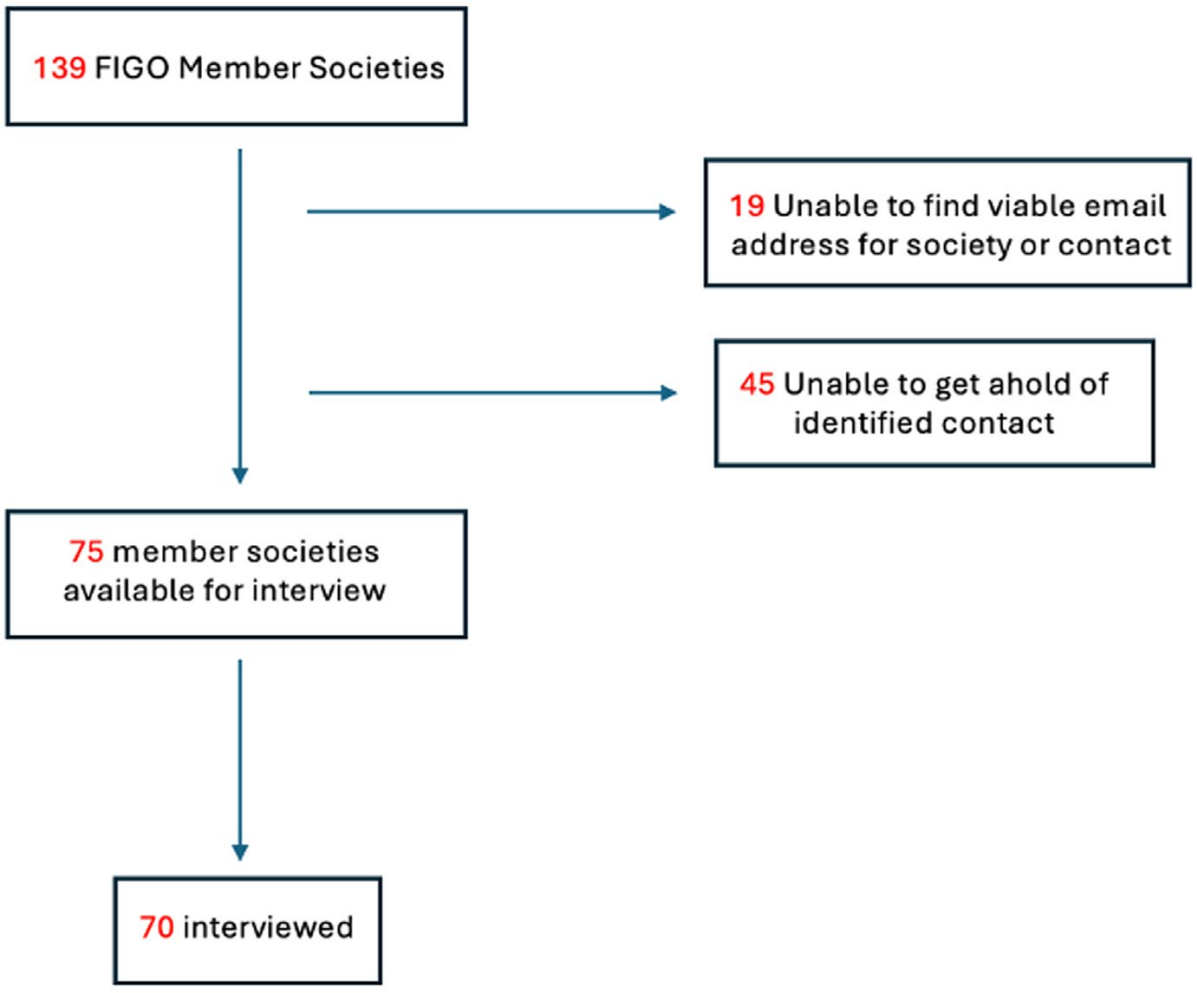
Flow chart of member society interview eligibility and participation.

**FIGURE 2:**
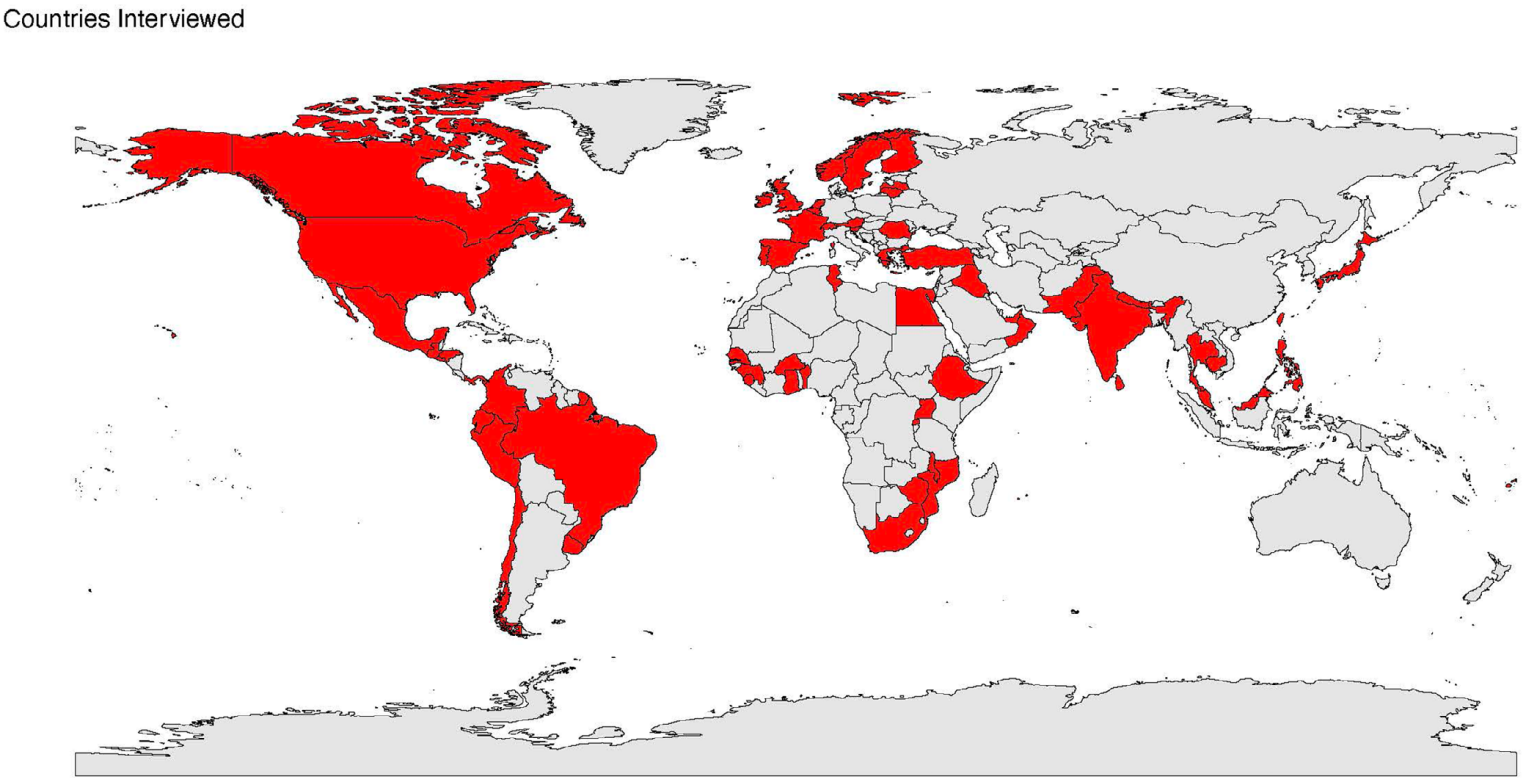
Global distribution of interviewed member societies.

Of the 139 FIGO member societies, individuals from 70 were interviewed (Figure 1). Member society participation was well‐distributed across all five FIGO regions (Table 1, Figure 2). Interviewees had spent an average of 14 years as members of their society and included general members to society presidents.

**TABLE 1:** Geographical representation of societies interviewed by FIGO region and FIGO Allied Regional Federations.

FIGO region	Member societies interviewed	Percentage of region interviewed *N* (%)
North America	USA Canada Mexico	3 (100%)
African Federation of Obstetrics and Gynecology (AFOG)	South Africa Malawi Zimbabwe Mozambique Sierra Leone Benin Burkina Faso Guinea Egypt Democratic Republic of Congo Ghana Ethiopia Ivory Coast Iraq Uganda United Arab Emirates Tunisia South Sudan Rwanda Oman Senegal	21 (47%)
Asia & Oceania Federation of Obstetrics and Gynecology (AOFOG)	Cambodia Japan Hong Kong Fiji Nepal Thailand Taiwan India Sri Lanka Singapore Papa New Guinea Philippines Pakistan Malaysia	14 (61%)
European Board and College of Obstetrics and Gynecology (EBCOG)	Portugal Lithuania Belgium France Greece Finland Austria Romania Latvia Macedonia Czech Republic UK Turkey Sweden Slovenia Norway Netherlands Ireland Switzerland Italy Spain	21 (62%)
Federation of Latin American Societies of Obstetrics and Gynecology (FLASOG)	Dominican Republic Brazil Chile Honduras Colombia Guatemala El Salvador Ecuador Uruguay Peru Panama	11 (61%)

**FIGURE 3:**
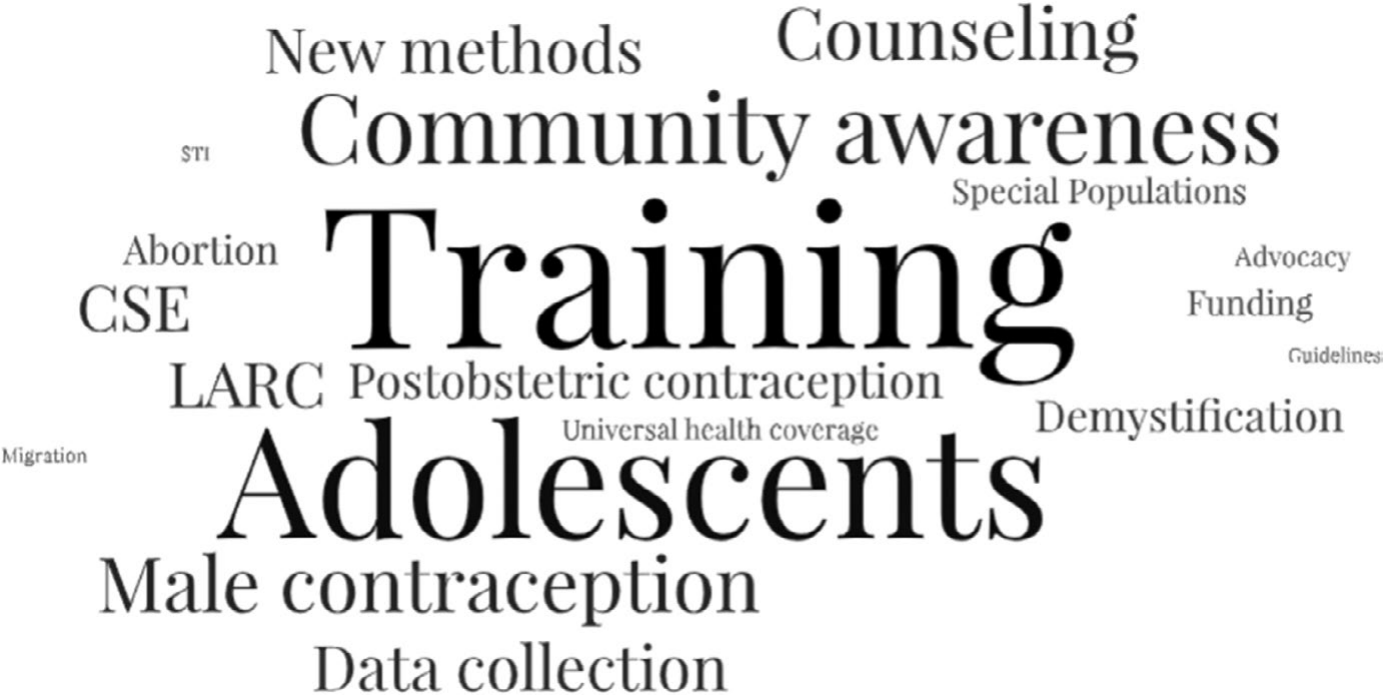
Word Cloud representing society priority areas for the next 5 years.

Among respondents, 19% indicated their society had a specific and dedicated working group or subcommittee focused on contraception with an additional 17% reporting a sexual and reproductive health (SRH) committee under which contraceptive programming was housed. Moreover, 84% of respondents indicated they had national contraceptive guidelines with the majority indicating that society members were involved in their development. Participants frequently reported training and capacity building, advocacy, community awareness, guideline development, educational workshops, and direct service delivery as areas of primary member society efforts—with healthcare worker training, adolescents, male contraception, community awareness, LARC's and comprehensive sexual education as future priority areas (Figure 3).

### 3.1 Healthcare worker (HCW) training

Despite the geographic diversity among participants, many reported a national shortage of reproductive healthcare experts and participants unanimously highlighted a critical training gap. When asked about contraceptive training, 59% stated they had some form of standardized national training although this ranged in implementation. For example, Nepal integrates family planning into pre‐service training for OBGYN residents, while the European Society for Contraception recently launched a new diploma in sexual and reproductive healthcare targeting OBGYNs, general practitioners, midwives, nurses and pharmacists.^14^ In other regions, training is less formalized, inconsistent, and often optional, relying on individual universities and personal initiatives. Several issues contribute to this including economic constraints, lack of comprehensive education in medical training, and a deprioritization of family planning in universities.

“Contraception is not a priority for many medical schools, and this is a vicious cycle; as many medical schools do not have hormonal IUDs, Mirena, Kyleena, and Implanon, they don't teach about the insertion of these methods.” (Latin America)

### 3.2 Public awareness and advocacy

Interviewees also discussed society engagement in advocacy and role in raising public awareness about reproductive health topics through mediums including talk shows, television or radio as well as working with celebrities and religious groups to reach the general public. Across platforms, the primary aims were often to address misinformation, inform women of their rights, and discuss abortion and contraceptive options, indications, and side effects.

“In all our public information, we emphasize that people have various choices. We present all ten contraceptive options for women and options for boys and men, such as sterilization and condom use. Our mission is to ensure that people can make well‐informed choices that best suit their needs and desires”. (Europe)

### 3.3 Adolescent contraception

Participants emphasized the need for the society to focus on comprehensive strategies to ensure adolescent access to contraception, including the need to prioritize LARCs. However, given cultural and religious contexts, it was reported that healthcare providers may not even offer appropriate counseling to adolescents or parents may not feel comfortable with adolescent contraceptive use. Adolescents may also lack awareness that they have the right to seek care.

“Our girls are getting pregnant and are also dying from at the ages of 10–14 years old… Abortions have also increased tremendously in adolescents, and that is a big challenge for us in contraception, because access, although efforts have been made, is very limited”. (Latin America)

Given the need, and corresponding challenges, several participants mentioned that their professional society actively strategizes to increase contraceptive coverage among adolescents with efforts ranging from advocacy, to promoting public awareness through digital media and peer‐to‐peer campaigns, to providing sex education in schools as part of school health programs to building adolescent‐specific clinics.

“A chunk of this is done through what are called school health programs. So, there is a complete health program in schools where people are trained to help adolescents regarding reproductive health and family planning issues and provide avenues for referrals as well.” (Africa)

“We also need to get a program on TV and radio telling women, especially adolescents, their rights to obtain contraception pills and the right to seek contraception in any place without parents.” (Latin America)

### 3.4 Local collaboration

Participants unanimously emphasized the need for the societies to encourage and facilitate collaboration across the health system—noting the importance of involving midwives, nurses, and general practitioners.

“[We launched] an integrated project combining post‐abortion care, safe abortion, and family planning. We trained healthcare providers from all public and private health facilities, working as a team.” (Africa)

Challenges to such collaboration include commitment and interest within the society itself ‐ with interviewees reporting varying levels of interest in family planning. A few noted that younger physicians are increasingly gravitating towards more lucrative surgical subspecialities and this gap is further amplified in countries struggling to retain physicians.

“The first time I was sent to go to the contraceptive training I was very upset because I thought that my boss was trying to undermine me. After I got trained into it, then I realized it's very very important. And the mindset that I had a lot of people still have it. When it comes to contraception, they think it's not important”. (Asia Oceania)

Furthermore, it is often difficult to get busy members to follow through on tasks and programs and initiatives often lose traction with funding, political will and leadership changes.

“Passion just changes with the leadership; it does not sustain”. (Asia Oceania)

### 3.5 International collaboration

**FIGURE 4:**
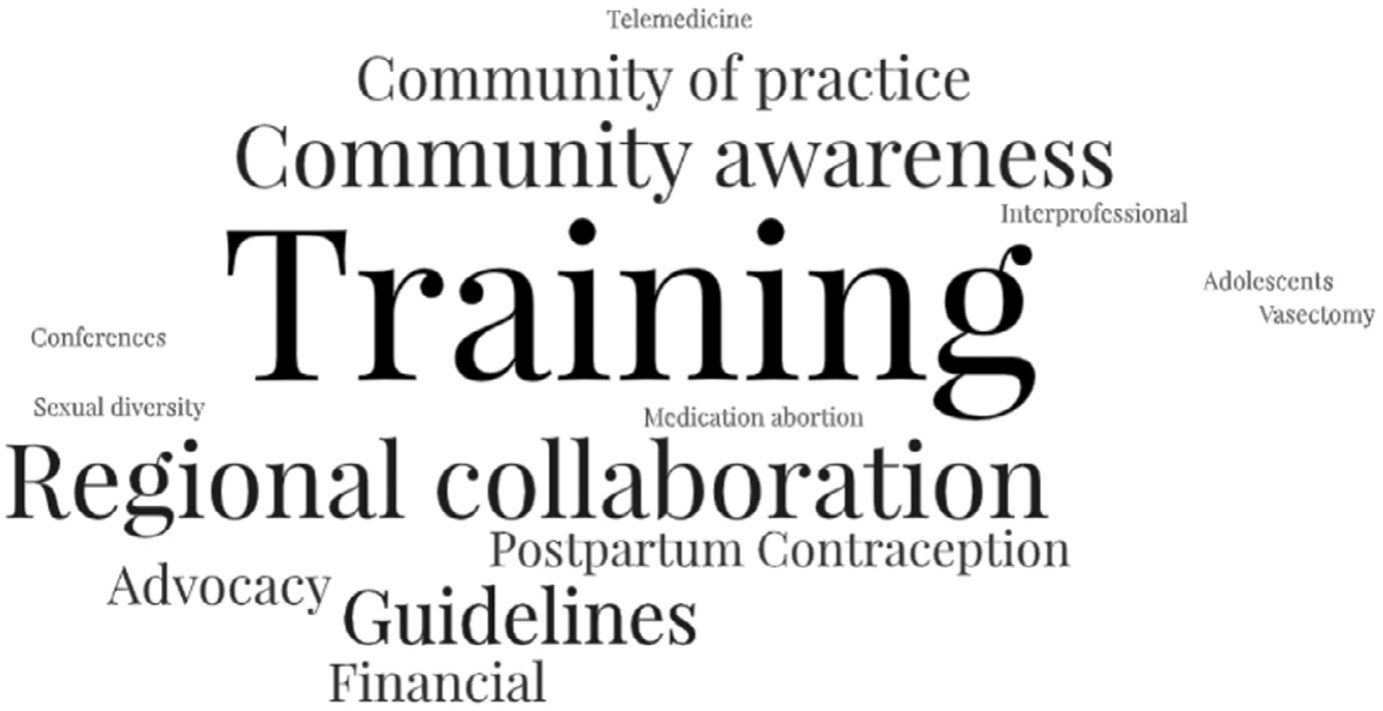
Word Cloud representing suggested areas for international collaboration.

On an international scale, participants expressed a desire for their society to collaborate with other member societies in efforts to train healthcare workers, develop guidelines, enhance community awareness and establish a community of practice (Figure 4). Given the varied time zones, languages, and differences in contraceptive landscape, a larger partnership, while helpful, respondents reported may be hard to operationalize. Local and regional collaboration were frequently highlighted as optimal avenues for cooperation.

“I find that sometimes when you're in your own space, and you're used to your working, your own country in your clinic and become very limited in what you could possibly do… But when you're on an international platform it just makes it so much easier for you to see beyond the work that you are doing. What other good can we do?”. (Europe)

Partnership with FIGO was also highlighted as adding value and credibility to the professional societies' work in‐country.

## 4 DISCUSSION

Maternal mortality remains one of the most pressing global issues with a dynamic and ever evolving landscape.^15^ While contraception is often overshadowed in favor of other sexual and reproductive health (SRHR) topics, efforts to increase contraceptive access across member societies were broad and diverse including training and capacity building, advocacy, community awareness, guideline development, research and direct service delivery. Broader contraceptive efforts included advocating for subsidized care or inclusion in universal health care, increased commodities purchasing, establishing family planning or youth clinics, and conducting nationwide surveys. Many interviewees also reflected on the influence and effectiveness of regional collaboration and the importance of working with those with similar cultural backgrounds or in close geographic proximity.

Challenges for societies remain large and constricting including lack of funding, reliable data, governmental policies, and a lack of interest from the private sector. Additionally, many societies reported resistance from religious authorities, affecting not only contraceptive uptake but also sexual education for adolescents and reproductive care for the LGBTQ+ community.

Furthermore, the interviews reveal a significant gap in public understanding of what contraception is and what it can do. To many respondents, it was increasingly clear that healthcare providers need to be better at tailoring contraceptive advice to meet the individual needs of patients and, at the same time, increasing public awareness, education, and individual empowerment. To do so, interviewees highlighted the importance of task sharing to extend the reach and effectiveness of family planning services and enhance collaboration and cooperation among stakeholders.

In light of these findings, there may be opportunities for member societies to identify contraceptive champions as part of a global network of contraceptive experts able to establish a best practices platform. Per the Cartagena declaration, each society is urged to form an SRHR division providing an opportunity to establish a global network under FIGO.^16^ Moreover, there may be opportunities for member societies, across all regions, to better integrate with international NGOs. For FIGO, there is a need to maintain a comprehensive and continually updated list of contact information for all member societies. Additional opportunities include encouraging intra‐ and inter‐regional collaboration, working to provide standardized pre‐service and in‐service educational materials, as well as materials to disseminate to the general public regarding contraceptive methods that are attuned to local and regional political, cultural, and religious norms.

### 4.1 Strengths and limitations

One of few studies assessing contraceptive programming across OBGYN member societies, this study's strength lies in its large regional representation and sample size. Additionally, conducting interviews across three languages allowed us to access a wide range of participants and perspectives.

This study did, however, have several limitations including the utilization of society members who may not know or represent the full extent of contraceptive work implemented within the society. To mitigate this, we requested representatives from the member society who were known to be the most involved and knowledgeable about SRHR work. Additionally, there were many countries for whom we were unable to interview in their primary language. This may have led to loss in interpretation of nuanced responses or misinterpretation around complex terminology meaning we may not have been able to get a true sense of the full scope of work or challenges faced by these societies.

## 5 CONCLUSION

Member societies face significant and diverse challenges globally in their work to address and improve contraceptive access, utilization, and safety. These include poor public awareness, cultural and religious stigma, and gaps in healthcare provider training. These barriers are shared, and interviewees consistently emphasized the role of governmental and international support, regional collaboration, funding and data as necessary to assist societies in advancing their work. OBGYN societies have the opportunity to bring a deep expertise and commitment to sexual and reproductive health. By understanding the landscape of their challenges, progress, and aspirations we, as an international community, can foster better communication, collaboration, and meaningful change in this field.

## AUTHOR CONTRIBUTIONS

Study conception: A.S. Development of methods: A.S., M.K. and P.R. Interviews: M.K., G.E. and M.R. Coding: M.K. and P.R. Analysis: A.S., M.K. and P.R. First draft of manuscript: M.K. Manuscript editing and revision: A.S., M.K. and P.R.

## FUNDING INFORMATION

Bill and Melinda Gates Foundation (Grant I049889).

## CONFLICT OF INTEREST STATEMENT

Aparna Sridhar, past chair of the FIGO Committee on Contraception.

## Supporting information


Data S1.

## Data Availability

Data available upon reasonable request.
