# Supplementary material for: The world of contraception: A global mapping project across FIGO member societies
Source: Int J Gynaecol Obstet. 2025 Jun 2;171(2):711–9. doi: 10.1002/ijgo.70254 (PMC12553095; doi:10.1002/ijgo.70254)
Supplement: Supplementary file 1 — Data S1. [file IJGO-171-711-s001.docx]

**Supplemental Documents:**

1. **Example of recruitment email**

I am reaching out on behalf of Dr. Aparna Sridhar, past Chair of the Committee on Contraception at FIGO. We are conducting a project funded by the Gates Foundation and supported by FIGO’s Committee on Contracpetion. This project is an effort to gain understanding and insight into the landscape of contraception programming across FIGO’s member soecities in an effort to help shape FIGO’s future collaborateive contraceptive work. We are wondering if someone form your member society would be interested in participating.

We are hoping to conduct a 60-minute interview to gain insight into your member societies’ work around contraception, the successes and challenges you have faced, and the opportunities you see for future collaboration in this space.

Can you please direct us to a personnel in your society who may be willing to participate in an interview? The interviews will be conducted via Zoom and will be audio recorded. If you would prefer the interview be in Spanish or French, pelase let us know and we can accommodate that request.

If you have any questions, please don’t hestitate to reach out. We appreciate your consideration.

1. **Interview Guide, English**

**Part A:**

To begin, we would like to start with some quick information about your member society
 1. What is your role in the member society?

 2. How many years have you been a part of the member society?

 3. Who is currently the president of your member society (at the time of interview)?

*Thank you for that information. I would like to shift gears and learn from you more about the work of your member society, focusing particularly on contraception. As a reminder, please think of this as a discussion and feel free to share any thoughts that come up, there are no right or wrong answers here. If you have any questions for me or need any clarifications, please don’t hesitate to ask.*

**PART B:**

4. Can you tell me what areas of obstetrics and gynecology or SRH services your member society works and focuses on?

5.  What are the highest priority areas for your society at this time? Where would you place contraceptive care on that priority list?

 5. Can you tell me about the contraceptive landscape in Ghana. What challenges/issues you may be facing or anticipating?

7. As you may know, some member societies have specific committees or working groups focused solely on contraception. We were wondering if your member society has any such groups, or is interested in starting one?

**Follow-up**
If yes committee: could you tell me more about the committee? [**probe**anything about the committee like how many members, how often they meet, what are the goals etc would be helpful

If no committee: Thank you for sharing. We know that everything looks different for different societies and that some have such groups and others do not.

·       I am wondering, does your society have working groups/committees on other topics?

·       Can you tell us about who the individuals are that work on contraception projects at your member society and what that looks like

8. A. Can you tell me about your experience with contraception guidelines? Does your society use any guidelines for your contraception work, for example, the WHO guidelines?
**Follow up:** If so, can you tell me more about how your society integrates these guidelines into your work?

B.    We also recognize that some countries have National Clinical Guidelines. We are curious to know if Ethiopia has national guidelines around contraceptive care, and if so, how your member society works with and incorporates those into your work.

C.     If working with MOH/governmental agencies hasn’t come up yet, Ask: What would you say is your working relationship with the MOH or similar governmental agencies? Do you see the member society as working closely with the private or public sector or both.

9. Can you please tell me about your experience with contraception training? For example, does your country have national training or qualifications in contraceptive care?

[ Probe: If Y, can you tell me more about it, duration of the training, what was it about, when were you trained. Follow up: are any members of your society national trainers or in any governmental working groups?]

**PART C**:

11.   A. For the next question, I am hoping you can think back on the last 5 years, say from 2018-2023. Have there been any projects your member society has been affiliated with directly related to contraception? We would love to hear about any sized project.  
 **[Probe:** ask for information about the project including funding, goals and project size/timeline]

B.    *If none of the projects mentioned were related to adolescents or postpartum*: can you think of projects that were specific to adolescents or postpartum family planning?

1. *If none of the projects mentioned were related to GBV/HIV/PAC*: can you think of projects that were specific to the integration of family planning with HIV, GBV PAC etc.?

12.One area we are interested in learning more about is whether your member society has been involved in any release of new contraceptive methods in your country. Can you tell me what the last new contraceptive that came onto the market in your country was and if your society has any experience or role within that? [**probe**: if there were any review meetings, or committees around the new contraceptive]

12. Looking into the next 5 years, can you please tell me what the field of contraceptive care looks like for your member society? Are there any programs or projects you are anticipating or hoping to engage in?

*Thank you for sharing your thought about the member society and priorities in contraception, next I have some questions around your experience particularly challenges, opportunities and collaborations.*

**PART D:**

13. Can you please tell me about your experience engaging with members on the issues of contraception and family planning?

14. Given the aim of this project is to find avenues for international collaboration, we would love to hear from you what might be some ways that collaboration with FIGO and other member societies would be most helpful to you? In what ways are they currently successful or unsuccessful? [**probe:** are there any goals/ vision that you have for collaboration with FIGO members in other countries? What would that look like for you]

15. Talking about collaboration with FIGO, it also conducts webinars/ events , has your society been able to engage/ participate in any such events organized my FIGO? If**Y:** What has your experience been like? any challenges that you face or any feedback? If**N:** can you tell me more about what has been most challenging in participating in these events? [**Probe:** is there anything that could have helped increase participation from your society like maybe a different time-zone, topic, or anything like that?]

10. Family planning 2030 is a global movement dedicated to advancing family planning rights. Are you familiar with this organization?

- Was your society involved in the governmental commitment to it?

1. **Interview Guide, French**

**Partie A :**

Nous aimerions commencer par quelques informations rapides sur votre société membre 
1. Quel est votre rôle dans la société membre ?

 2. Depuis combien d'années faites-vous partie de la société membre ?

 3. Qui est actuellement le président de votre association membre (au moment de l'entretien) ?

**PARTIE B :** 
4. Pouvez-vous me parler brièvement du paysage contraceptif dans votre pays ? [**Aller plus loin** : Quels sont, selon vous, les plus grands problèmes actuels ou à venir dans le domaine de la contraception dans votre région ?

5. Sur quels domaines votre société membre se concentre-t-elle ?

6. Il semble que vous travailliez sur de nombreux domaines, y compris. Si vous deviez choisir les domaines les plus prioritaires pour votre société à l'heure actuelle, quels seraient-ils ? Et comme nous souhaitons en savoir plus sur la contraception en particulier, où placeriez-vous les soins contraceptifs dans cette liste de priorités ?

[**Aller plus loin** : Pouvez-vous nous dire quelles sont vos priorités en tant qu'organisation et où se situerait la contraception dans cette liste de priorités ?]

7. Comme vous le savez peut-être, certaines sociétés membres ont des comités ou des groupes de travail spécifiques dédiés à la contraception. Votre société membre dispose-t-elle de tels groupes ou est-elle intéressée par la création d'un tel groupe ?

**Poursuivre :** 
Si oui, un comité : Pourriez-vous m'en dire plus sur le comité ? [**Allez plus loin** sur tout ce qui concerne le comité, comme le nombre de ses membres, la fréquence de ses réunions, ses objectifs, etc.]

Si non, pas de comité : Merci pour votre réponse. Nous savons que les choses se présentent différemment selon les sociétés et que certaines ont de tels groupes et d'autres non.

- Je me demandais, votre société a-t-elle des groupes de travail/comités sur d'autres sujets ?
- Pouvez-vous nous dire qui sont les personnes qui travaillent sur les projets de contraception au sein de votre société membre et à quoi cela ressemble ?

8. A. Pouvez-vous me parler de votre expérience en matière de recommandations sur la contraception ? Votre société utilise-t-elle des recommandations pour votre travail sur la contraception, par exemple les recommandations de l'OMS ? 
**Poursuivre :** Si c’est le cas, pouvez-vous m'en dire plus sur la manière dont votre société intègre ces recommandations dans votre travail ?  

1. Nous reconnaissons également que certains pays disposent de recommandations cliniques nationales. Nous sommes curieux de savoir si {insérer le nom du pays} dispose de recommandations nationales en matière de contraception et, dans l'affirmative, comment votre société membre travaille avec ces recommandations et les intègre dans son travail.
2. Si la question de la collaboration avec le ministère de la Santé ou les agences gouvernementales n'a pas encore été abordée, posez la question suivante : Quelles sont vos relations de travail avec le ministère de la Santé ou les agences gouvernementales similaires ? Considérez-vous que l'association membre travaille en étroite collaboration avec le secteur privé, le secteur public ou autre ?

9. Pouvez-vous me parler de votre expérience en matière de formation à la contraception ? Par exemple, votre pays dispose-t-il d'une formation ou de qualifications nationales en matière de contraception ?

[ **Aller plus loin** : Si oui, pouvez-vous m'en dire plus à ce sujet, sur la durée de la formation, sur quoi elle portait, quand avez-vous été formé ? Suivi : certains membres de votre société sont-ils des instructeurs nationaux ou font-ils partie d'un groupe de travail gouvernemental ?]

10. Planning familial 2030 (FP2030) est un mouvement mondial qui se consacre à l'avancement des droits en matière de planning familial. Connaissez-vous cette organisation ?

**Poursuivre :**

Si le pays s'est déjà engagé dans FP2030 : Il semble que votre pays se soit déjà engagé dans FP2030. Votre société professionnelle est-elle au courant de cet engagement et de ce qu'il implique ?

Si le pays ne s'est pas engagé dans le FP2030 : Pensez-vous qu'il y aurait un intérêt au sein de votre société à en savoir plus sur les objectifs de 2030 ?

**PARTIE C** :  

11.   A. Pour la question suivante, je vous invite à réfléchir aux cinq dernières années, disons de 2018 à 2023. Votre société membre a-t-elle participé à des projets directement liés à la contraception ? Nous serions ravis d'entendre parler de projets de toute taille.

Si **Oui**, cela paraît être un travail incroyablement intéressant et important, merci. Quelques questions rapides de suivi spécifiques à chaque projet :

- Quel était le titre du projet ?
- Pouvez-vous m'en dire plus, notamment sur son champ d'action (national, local) et ses groupes cibles ?
- Quand avez-vous commencé le projet (calendrier), bailleur de fonds, objectifs.

1. *Si aucun des projets mentionnés n'était lié aux adolescents ou au post-partum* : Comme vous le savez sans doute, le planning familial pour le post-partum et les soins de santé reproductive pour les adolescents sont devenus une priorité croissante au niveau international, en particulier pour des organisations telles que la FIGO, l'OMS et l'UNESCO. Ces domaines ont été identifiés comme des lacunes potentielles dans les soins et comme des domaines importants pour les travaux et les investissements futurs dans le but de fournir des soins de santé reproductive pour tous complets, en temps utile et souhaités. 
    
   À ce titre, nous nous demandions si, au cours des cinq dernières années, il y avait eu dans votre société des projets axés spécifiquement sur le planning familial du post-partum ou sur les adolescents.

[**Aller plus loin** : Si **Oui**, pouvez-vous m'en dire plus sur ces projets ? De quoi s'agissait-il, quels étaient les objectifs, le financement, le calendrier, etc.] 

1. *Si aucun des projets mentionnés n'est lié à VBG/VIH/Soins post-avortement* : Dans le même ordre d'idées, dans certaines circonstances, le travail sur la contraception est intégré dans le dépistage ou les programmes liés à d'autres thèmes de santé reproductive tels que la violence liée au genre, le VIH et les soins post-avortement. Votre société membre a-t-elle participé à des projets ou programmes de ce type ?

1. Nous aimerions savoir si votre société membre a participé à la mise sur le marché de nouvelles méthodes contraceptives dans votre pays. Pouvez-vous me dire quel a été le dernier nouveau contraceptif mis sur le marché dans votre pays et si votre société a une expérience ou un rôle dans ce domaine ? [**Aller plus loin** : Par exemple, s'il y a eu des réunions d'examen ou des comités autour du nouveau contraceptif, j'aimerais en savoir plus sur la manière dont votre société membre a été impliquée dans ce processus.]

12. Pour les cinq prochaines années, pouvez-vous me dire à quoi ressemble le domaine des soins contraceptifs pour votre société membre ? Y a-t-il des programmes ou des projets que vous prévoyez ou espérez mettre en œuvre ?

**PARTIE D** :

13. Pouvez-vous me parler de votre expérience en matière d'engagement avec les membres sur les questions de contraception et de planning familial ? Vous avez mentionné [citer les défis qu'ils ont soulevés], pouvez-vous me dire comment cela s'est passé ?

14. Étant donné que l'objectif de ce projet est de trouver des pistes de collaboration internationale, nous serions ravis de savoir comment la collaboration avec la FIGO et d'autres sociétés membres pourrait vous être le plus utile. De quelle manière sont-elles actuellement efficaces ou inefficaces ?

[ **Aller plus loin** : Avez-vous des objectifs/une vision de la collaboration avec les membres de la FIGO dans d'autres pays ? À quoi cela ressemblerait-il pour vous ?]

15. En ce qui concerne la collaboration avec la FIGO, celle-ci organise également des webinaires/événements. Votre société a-t-elle pu s'engager/participer à l'un de ces événements organisés par la FIGO ? Si **Oui** : Quelle a été votre expérience ? Des difficultés rencontrées ou un retour d'information ? Si **Non** : pouvez-vous m'en dire plus sur ce qui a été le plus difficile dans la participation à ces événements ? [**Aller plus loin** : quelque chose aurait pu aider à augmenter la participation de votre société, comme par exemple un fuseau horaire différent, un sujet différent, ou un autre facteur de ce genre].

1. **Interview Guide, Spanish**

**Part A:**

Me gustaría empezar preguntándole algunos datos sobre su organización: 
1. ¿Cuál es su función en la organización?

 2. ¿Cuántos años lleva formando parte de esta organización?

 3. ¿Quién es actualmente el presidente de su organización (en el momento de esta entrevista)?

**PART B:** 

4. ¿Puede hablarme brevemente del panorama actual de la anticoncepción en su país? [**Profundizar**: ¿cuáles cree que son los principales problemas actuales o futuros en materia de anticoncepción para su región?]

5. ¿Cuáles son las áreas de interés de su organización?

6. Da la impresión de que hay muchas áreas en las que están trabajando, entre ellas {resuma su respuesta}. Si tuviera que elegir las áreas más prioritarias para su organización en este momento, ¿cuáles serían? Y, dado que estamos interesados en saber más sobre la anticoncepción en particular, ¿dónde situaría la atención anticonceptiva en esa lista de prioridades? 
 
[**Profundizar:** ¿Puede decirnos cuáles son sus prioridades principales como organización y dónde encajaría la anticoncepción en esa lista de prioridades?] 

7. Como quizá sepa, algunas organizaciones cuentan con comités o grupos de trabajo específicos centrados exclusivamente en la anticoncepción. Nos preguntamos si su organización cuenta con algún grupo de este tipo, o si está interesada en crear uno. 
 
**Follow-up:** 
En caso de sí tener comité: ¿podría contarme más sobre el comité? [indagar sobre el comité, como cuántos miembros, con qué frecuencia se reúnen, cuáles son los objetivos, etc.]

Si no tiene comité: Gracias por compartir. Sabemos que cada organización es distinta y que algunas tienen grupos de este tipo y otras no.

- ¿Tiene su organización grupos de trabajo/comités sobre otros temas?
- ¿Podría decirnos quiénes son las personas que trabajan en proyectos de anticoncepción en su organización y qué tipo de trabajo realizan?

8. A. ¿Podría hablarme de su experiencia con las pautas para el trabajo anticonceptivo? ¿Utiliza su organización alguna guía para realizar el trabajo de anticoncepción, por ejemplo, las pautas de la OMS?

**Seguimiento:** En caso afirmativo, ¿puede contarme más sobre cómo integra su organización estas pautas en su trabajo? 

1. También reconocemos que algunos países cuentan con pautas clínicas nacionales. Tenemos curiosidad por saber si {inserte el nombre del país} dispone de pautas nacionales en torno a la atención anticonceptiva y, de ser así, cómo trabaja su organización con ellas y cómo las incorpora a su trabajo.
2. Si aún no ha surgido el tema de trabajar con el ministerio de salud/organismos gubernamentales, pregunte: ¿Cuál diría que es su relación de trabajo con el ministerio de salud u otros organismos gubernamentales similares? ¿Considera que su organización trabaja más de cerca con el sector privado, el sector público u otros?

9. ¿Podría hablarme de su experiencia con la capacitación para la educación anticonceptiva? Por ejemplo, ¿dispone su país de formaciones o capacitaciones nacionales en materia de anticoncepción?

[**Profundizar**: Si la respuesta es sí, ¿podría contarme más sobre ello, como cuánto dura la formación, de qué trata y cuándo recibió la formación? **Seguimiento**: ¿algún miembro de su sociedad es formador nacional o está en algún grupo de trabajo gubernamental]?

10. Planificación familiar 2030 es un movimiento mundial dedicado a promover los derechos en materia de planificación familiar. ¿Conoce esta organización?

**Seguimiento**

Si el país ya se ha comprometido con FP2030: parece que su país ya se ha comprometido con la FP2030, ¿está su organización enterada de esto y de lo que implica el compromiso?

Si el país no se ha comprometido con la FP2030: ¿Cree que habría interés en su organización por saber más sobre los objetivos para el 2030?

**PART C**:  

11.   A. Para la siguiente pregunta, quisiera que pensara en últimos 5 años, digamos que del 2018 al 2023. ¿Ha habido algún proyecto en el que su organización haya estado afiliada directamente relacionado con la anticoncepción? Nos encantaría saber sobre proyectos de cualquier tamaño.

**En caso afirmativo:** suena como un trabajo increíblemente interesante e importante, gracias. Un par de preguntas rápidas de seguimiento específicas para cada proyecto...

- ¿Cuál era el título del proyecto?
- ¿Puede contarme más sobre él, por ejemplo, el alcance (nacional, local) y los grupos a los que iba dirigido?
- ¿Cuándo inició el proyecto, quién lo financió, cuáles eran los objetivos?

1. *Si ninguno de los proyectos mencionados está relacionado con adolescentes o el postparto:* Como probablemente sepa, la planificación familiar postparto y los cuidados de salud reproductiva de las adolescentes se han convertido en una prioridad en el espacio internacional, especialmente para organizaciones como FIGO, la OMS y UNESCO. Estas áreas han sido identificadas como posibles lagunas en la atención y áreas importantes para futuros trabajos e inversiones con el objetivo de proporcionar una atención reproductiva integral, oportuna y justa para todos. 
    
   Por ello, queremos saber si en los últimos cinco años ha habido en su organización algún proyecto centrado específicamente en la planificación familiar postparto o para adolescentes.

[**Profundizar**: En caso afirmativo**,** ¿puede hablarme más de estos proyectos? De qué trataban, objetivos, financiación, duración, etc.].

1. *Si ninguno de los proyectos mencionados estaba relacionado con la violencia de género, el VIH o la atención tras un aborto:* Algunas veces el trabajo con anticonceptivos se integra con los esfuerzos de detección o en programas relacionados con otros temas de salud reproductiva como la violencia de género, el VIH y la atención postaborto. ¿Ha participado su organización en algún proyecto o programa de este tipo?
2. Un área sobre la que estamos interesados en saber más es si su organización ha participado en algún lanzamiento de nuevos métodos anticonceptivos en su país. ¿Puede decirme cuál fue el último nuevo anticonceptivo que salió al mercado en su país y si su organización tiene alguna experiencia o papel en ello? [**profundizar**: por ejemplo, si hubo alguna reunión de revisión, o comités en torno al nuevo anticonceptivo, me encantaría saber más sobre cómo participó su organización en este proceso].

12. ¿Podría decirme cómo se ve el campo de la anticoncepción para su organización de cara a los próximos 5 años? ¿Hay algún programa o proyecto que prevea o en el que espere participar?

**PART D**:

13. ¿Podría hablarme de su experiencia a la hora de trabajar con los miembros en temas de anticoncepción y planificación familiar? Usted mencionó [reflexione sobre los retos que han planteado] ¿podría decirme cómo fue eso? 

14. Dado que el objetivo de este proyecto es encontrar vías de colaboración internacional, nos encantaría que nos contara cuáles podrían ser algunas de las formas en las que una colaboración con FIGO y otras organizaciones le podrían resultar útiles. ¿En qué aspectos tiene éxito o no su organización actualmente? 
[**profundizar**: ¿hay algún objetivo/visión que tenga para la colaboración con los miembros de FIGO en otros países? ¿Cómo sería?]

15. Hablando de la colaboración con FIGO, ésta también lleva a cabo seminarios web/eventos, ¿ha podido su organización participar en alguno de estos eventos organizados por FIGO? Si la respuesta es **sí:** ¿cómo ha sido su experiencia? ¿algún reto al que se haya enfrentado o algún comentario? Si la respuesta es **no**: ¿puede decirme más sobre lo que ha supuesto un mayor reto a la hora de participar en estos eventos? [Profundizar: ¿hay algo que podría haber ayudado a aumentar la participación de su organización, como por ejemplo un horario diferente, un tema o algo por el estilo?]

1. **Code Book**

| **Id** | **Parent Id** | **Depth** | **Title** |
| --- | --- | --- | --- |
| 1 |  | 0 | RQ: Collaboration |
| 2 | 1 | 1 | Challenges in collaboration (external) |
| 3 | 1 | 1 | Collaboration with other NGOs and agencies |
| 4 | 1 | 1 | Collaborations with wider health systems |
| 5 | 1 | 1 | Govt entities |
| 6 | 5 | 2 | Aligned with government: integration of FP into PP and PAC |
| 7 | 5 | 2 | Serve as technical expert and advisor |
| 8 | 1 | 1 | Independent from gov |
| 9 | 1 | 1 | Interest in collaboration with other member societies and countries |
| 10 | 1 | 1 | Need for formal collaborations with wider health systems |
| 11 |  | 0 | Discuss with coder |
| 12 |  | 0 | Innovation to enhance care |
| 13 |  | 0 | Regroup later |
| 14 | 13 | 1 | Good quote |
| 16 |  | 0 | RQ: AREAS of work; member society |
| 17 | 16 | 1 | Advocacy |
| 18 | 16 | 1 | Awareness and community engagement |
| 19 | 16 | 1 | Non hormonal treatments |
| 20 | 16 | 1 | Not focused in contraception |
| 21 | 16 | 1 | Organize conferences and workshops |
| 22 | 16 | 1 | Research |
| 23 | 16 | 1 | Service delivery |
| 24 | 23 | 2 | Comprehensively improving women's' health |
| 25 | 23 | 2 | Patient engagement and counselling |
| 26 | 16 | 1 | Training and capacity building |
| 27 | 16 | 1 | Works closely with adolescents |
| 28 | 16 | 1 | Writing guidelines |
| 29 |  | 0 | RQ: Collaboration with FIGO |
| 30 | 29 | 1 | FIGO can provide contraception training to GPs |
| 31 | 29 | 1 | FIGO's influence, credibility |
| 32 | 29 | 1 | Help improve healthcare initiatives |
| 33 | 29 | 1 | Help in managing commodities |
| 34 | 29 | 1 | Past collaborations with FIGO |
| 35 | 29 | 1 | RQ: FIGO webinars |
| 36 | 35 | 2 | Interested in direct engagement/organize webinars |
| 37 | 35 | 2 | Not participated in FIGO webinars |
| 38 | 35 | 2 | Participated in webinars |
| 39 | 35 | 2 | Suggestions challenges about webinars |
| 40 | 35 | 2 | Unclear about the stance in webinar |
| 41 | 35 | 2 | Webinar topics |
| 42 | 29 | 1 | Strengthen collaboration and alliance (external) |
| 43 |  | 0 | RQ: Contraceptive guidelines |
| 44 | 43 | 1 | Guidelines References |
| 45 | 44 | 2 | FIGO guidelines |
| 46 | 44 | 2 | SRH guidelines |
| 47 | 44 | 2 | Royal college guidelines |
| 48 | 44 | 2 | WHO guidelines |
| 49 | 43 | 1 | Have national guidelines |
| 50 | 43 | 1 | No national guidelines |
| 51 | 43 | 1 | Refer to international guides and adapt to local needs |
| 52 | 43 | 1 | Standardized guidelines but not updated recently |
| 53 | 43 | 1 | Unclear if there is a standardized system |
| 54 |  | 0 | RQ: contraceptive LANDSCAPE in the country |
| 55 | 54 | 0 | RQ: Challenges |
| 56 | 55 | 2 | Cultural and religious barriers |
| 57 | 55 | 2 | Factors related to govt policies, practices |
| 58 | 58 | 3 | Concerns about pills and prescription |
| 60 | 58 | 3 | Gaps in training among HCW |
| 61 | 58 | 3 | Lack of standardized training and guides |
| 62 | 58 | 3 | Shortage of healthcare workforce |
| 63 | 55 | 2 | Inequity in contraceptive provision and access |
| 65 | 55 | 2 | Lack of basic infrastructures for contraceptive care |
| 66 | 55 | 2 | Other challenges |
| 67 | 54 | 1 | Contraceptive methods: availability and uptake |
| 68 | 67 | 2 | Available methods |
| 69 | 67 | 2 | increasing focus and use |
| 70 | 67 | 2 | Low uptake for available methods |
| 71 | 67 | 2 | Methods widely used |
| 72 | 67 | 2 | Unavailable |
| 73 | 54 | 1 | Current strategies |
| 74 | 73 | 2 | Free and subsidized care |
| 75 | 73 | 2 | Increasing focus on pt engagement and counselling |
| 76 | 73 | 2 | Task sharing collaboration and capacity building |
| 77 | 54 | 1 | Different entities |
| 78 | 54 | 1 | Government efforts |
| 79 | 54 | 1 | HCWs and their roles |
| 80 | 79 | 2 | GP roles |
| 81 | 79 | 2 | Midwives roles |
| 82 | 79 | 2 | Nurses roles |
| 83 | 79 | 2 | OBGYN roles |
| 84 | 79 | 2 | Pharmacists |
| 85 | 54 | 1 | High abortion rates |
| 86 | 54 | 1 | Issues in adol care |
| 87 | 86 | 2 | Decrease rate of adolescent pregnancies |
| 88 | 86 | 2 | Education and awareness |
| 89 | 86 | 2 | Negative health outcomes |
| 90 | 54 | 1 |  |
| 91 | 54 | 1 | Positive health outcomes |
| 92 | 91 | 2 | Awareness about health benefits side effects |
| 94 |  | 0 | RQ: Contraceptive Training |
| 95 | 94 | 1 | MEC wheel |
| 96 | 94 | 1 | No standardized training |
| 97 | 94 | 1 | Part of medical course |
| 98 | 94 | 1 | Standardized training |
| 99 | 94 | 1 | Unclear if training is standardized |
| 100 |  | 0 | RQ: contribution in RELEASE of new medications |
| 101 | 100 | 1 | High engagement for in use of new contraceptive methods |
| 102 | 100 | 1 | Low engagement in new releases |
| 103 | 100 | 1 | New meds |
| 104 | 100 | 1 | No engagement in meds release |
| 105 | 100 | 1 | No recent releases |
| 106 |  | 0 | RQ: Familiarity with FP2030 |
| 107 | 106 | 1 | Aware about the FP2030 |
| 108 | 106 | 1 | Not aware about FP 2030 and interested to learn |
| 109 |  | 0 | RQ: funding sources |
| 110 | 109 | 1 | Funded by pharma and industry |
| 111 | 109 | 1 | No state funding |
| 112 | 109 | 1 | Priorities change with funding |
| 113 |  | 0 | RQ: INTEGRATION of contraceptive care |
| 114 | 113 | 1 | Adolescents and contraceptive care |
| 115 | 114 | 2 | Adol focused services |
| 116 | 114 | 2 | Adol: education and awareness focused integration |
| 117 | 114 | 2 | Increased funding for adol contraception care |
| 118 | 114 | 2 | Not engaged with aldols SRH |
| 119 | 113 | 1 | Difficulty to integrate contraception, HIV and abortion care due to changing legal abortion laws |
| 120 | 113 | 1 | Fragmented care; no integration |
| 121 | 113 | 1 | GBV and contraception integrated |
| 122 | 121 | 2 | GBV not integrated |
| 123 | 113 | 1 | HIV and contraception |
| 124 | 123 | 2 | Awareness in HIV along with other STIs |
| 125 | 123 | 2 | HIV not integrated |
| 126 | 113 | 1 | LGBTQA |
| 127 | 113 | 1 | Post-partum contraception |
| 128 | 127 | 2 | No program on post-partum contraception |
| 129 |  | 0 | RQ: interviewee's ROLE & RESPONSIBILITY |
| 130 | 129 | 1 | Cofounders |
| 131 | 129 | 1 | Leadership |
| 132 | 129 | 1 | Non- leadership |
| 133 |  | 0 | RQ: Member society engagement |
| 134 | 133 | 1 | Challenge to society implementation |
| 135 | 133 | 1 | Challenges within member society |
| 136 | 133 | 1 | No challenges collaborating within the society |
| 137 |  | 0 | RQ: PRIORITIES for the member society |
| 138 | 137 | 1 | Contraception not the main priority |
| 139 | 137 | 1 | Expand and improve service delivery |
| 140 | 139 | 2 | Contraception in adols and youths |
| 141 | 137 | 1 | Improve research and collab |
| 142 | 137 | 1 | Recruitment and retention |
| 143 | 137 | 1 | Top priority |
| 144 | 137 | 1 | Writing guidelines |
| 145 |  | 0 | RQ: projects in the NEXT FIVE years |
| 146 | 145 | 1 | Advance collaboration, partnership |
| 147 | 145 | 1 | Capacity building, education and training |
| 148 | 145 | 1 | Expand services and access to care |
| 149 | 145 | 1 | Focus on adolescent care |
| 150 | 145 | 1 | Improve counselling and pts engagement |
| 151 | 145 | 1 | Integrate technology |
| 152 | 145 | 1 | NDI project with FIGO |
| 153 | 145 | 1 | No specific projects |
| 154 |  | 0 | RQ: projects in the PAST FIVE years |
| 155 | 154 | 1 | Contraceptive programs |
| 156 | 154 | 1 | Counselling and patient engagement |
| 157 | 154 | 1 | Education and training |
| 158 | 154 | 1 | Programs affected by conflict (broader context) |
| 159 | 154 | 1 | Research and publication |
| 160 |  | 0 | RQ: YEARS experience in the role |
| 161 | 160 | 1 | 10 to 15 years with the member society |
| 162 | 160 | 1 | 15 to 20 years with the member society |
| 163 | 160 | 1 | 5 to10 years with member society |
| 164 | 160 | 1 | Less than 5 years with the society |
| 165 | 160 | 1 | More than 20 years with member society |
| 167 |  | 0 | Working groups |
| 168 | 167 | 1 | Have working groups for FP |
| 169 | 168 | 2 | Ad hoc meetings |
| 170 | 168 | 2 | Structured, frequent meetings |
| 171 | 167 | 1 | No working group for contraception |
| 172 | 167 | 1 | No working groups for any reproductive health topics |

(6)

COREQ (COnsolidated criteria for REporting Qualitative research) Checklist

A checklist of items that should be included in reports of qualitative research. You must report the page number in your manuscript where you consider each of the items listed in this checklist. If you have not included this information, either revise your manuscript accordingly before submitting or note N/A.

| **Topic** | **Item No.** | **Guide Questions/Description** | **Reported on**  **Page No.** |
| --- | --- | --- | --- |
| **Domain 1: Research team**  **and reﬂexivity** | | | |
| *Personal characteristics* | | | |
| Interviewer/facilitator | 1 | Which author/s conducted the interview or focus group? | 4 |
| Credentials | 2 | What were the researcher’s credentials? E.g. PhD, MD | 4 |
| Occupation | 3 | What was their occupation at the time of the study? | 4 |
| Gender | 4 | Was the researcher male or female? | 4 |
| Experience and training | 5 | What experience or training did the researcher have? | 4 |
| *Relationship with*  *participants* | | | |
| Relationship established | 6 | Was a relationship established prior to study commencement? | 4 |
| Participant knowledge of  the interviewer | 7 | What did the participants know about the researcher? e.g. personal  goals, reasons for doing the research |  |
|  |  |  | 4 |
|  |  |  |  |
| Interviewer characteristics | 8 | What characteristics were reported about the inter viewer/facilitator?  e.g. Bias, assumptions, reasons and interests in the research topic |  |
|  |  |  | 4 |
|  |  |  |  |
| **Domain 2: Study design** | | | |
| *Theoretical framework* | | | |
| Methodological orientation and Theory | 9 | What methodological orientation was stated to underpin the study? e.g. grounded theory, discourse analysis, ethnography, phenomenology,  content analysis |  |
|  |  |  | 5 |
|  |  |  |  |
| *Participant selection* | | | |
| Sampling | 10 | How were participants selected? e.g. purposive, convenience,  consecutive, snowball |  |
|  |  |  | 4 |
|  |  |  |  |
| Method of approach | 11 | How were participants approached? e.g. face-to-face, telephone, mail,  email |  |
|  |  |  | 4 |
|  |  |  |  |
| Sample size | 12 | How many participants were in the study? | 4 |
| Non-participation | 13 | How many people refused to participate or dropped out? Reasons? | 4 |
| *Setting* | | | |
| Setting of data collection | 14 | Where was the data collected? e.g. home, clinic, workplace | 5 |
| Presence of non-  participants | 15 | Was anyone else present besides the participants and researchers? |  |
|  |  |  | 5 |
|  |  |  |  |
| Description of sample | 16 | What are the important characteristics of the sample? e.g. demographic  data, date |  |
|  |  |  | 4 |
|  |  |  |  |
| *Data collection* | | | |
| Interview guide | 17 | Were questions, prompts, guides provided by the authors? Was it pilot  tested? | 5 |
|  |  |  |  |
| Repeat interviews | 18 | Were repeat inter views carried out? If yes, how many? | 5 |
| Audio/visual recording | 19 | Did the research use audio or visual recording to collect the data? | 5 |
| Field notes | 20 | Were ﬁeld notes made during and/or after the inter view or focus group? | 5 |
| Duration | 21 | What was the duration of the inter views or focus group? | 5 |
| Data saturation | 22 | Was data saturation discussed? | 5 |
| Transcripts returned | 23 | Were transcripts returned to participants for comment and/or | 5 |

| **Topic** | **Item No.** | **Guide Questions/Description** | **Reported on**  **Page No.** |
| --- | --- | --- | --- |
|  |  | correction? |  |
| **Domain 3: analysis and**  **ﬁndings** | | | |
| *Data analysis* | | | |
| Number of data coders | 24 | How many data coders coded the data? | 5 |
| Description of the coding  tree | 25 | Did authors provide a description of the coding tree? |  |
|  |  |  | Supplemental |
|  |  |  |  |
| Derivation of themes | 26 | Were themes identiﬁed in advance or derived from the data? | 5 |
| Software | 27 | What software, if applicable, was used to manage the data? | 5 |
| Participant checking | 28 | Did participants provide feedback on the ﬁndings? | 6 |
| *Reporting* | | | |
| Quotations presented | 29 | Were participant quotations presented to illustrate the themes/ﬁndings?  Was each quotation identiﬁed? e.g. participant number |  |
|  |  |  | 6-8 |
|  |  |  |  |
| Data and ﬁndings consistent | 30 | Was there consistency between the data presented and the ﬁndings? | 6-8 |
| Clarity of major themes | 31 | Were major themes clearly presented in the ﬁndings? | 6-8 |
| Clarity of minor themes | 32 | Is there a description of diverse cases or discussion of minor themes? | 6-8 |

Developed from: Tong A, Sainsbury P, Craig J. Consolidated criteria for reporting qualitative research (COREQ): a 32-item checklist for interviews and focus groups. *International Journal for Quality in Health Care*. 2007. Volume 19, Number 6: pp. 349 – 357

**Once you have completed this checklist, please save a copy and upload it as part of your submission. DO NOT include this checklist as part of the main manuscript document. It must be uploaded as a separate file.**
